# Chondroitin sulfate protects against synaptic impairment caused by fluorosis through the Erk1/2-MMP-9 signaling pathway

**DOI:** 10.1038/s41598-025-14631-7

**Published:** 2025-08-13

**Authors:** Fujun Ai, Shengyuan Wang, Ling Ye, Wen Wan, Xiao Zhou, Minghai Liu, Kaiju Mo, Yongheng Lu, Na Wei, Zhizhong Guan, Yanjie Liu

**Affiliations:** 1 Department of Pathology, The Affiliated Hospital of Guizhou Medical University, Guiyang, 550004 China; 2 Pathology Morphology and Molecular Laboratory of the Affiliated Hospital of Guizhou Medical University, Guiyang, 550004 China; 3 Department of Pathology, Lin Yi People’s Hospital, Linyi, Shandong China; 4 Department of Neurology, The People’s Hospital of Huaxi District, Guiyang, 550025 China; 5 Department of Pathology, Guizhou Medical University, Guiyang, 550004 Guizhou China; 6 Key Laboratory of Endemic and Ethnic Diseases of the Ministry of Education, Guizhou Medical University, Guiyang, 550004 China

**Keywords:** Fluoride, Chondroitin sulfate, Erk1/2, MMP-2, MMP-9, SYN, Oncogenesis, Brain injuries

## Abstract

Prolonged exposure to fluoride may induce neurotoxic effects. Chondroitin sulfate (CS) exhibits protective functions within the central nervous system (CNS); however, the mechanism by which CS protects synapses against fluoride remains incompletely understood. Our objective was to investigate the protective efficacy of CS on synapses and decipher its underlying mechanisms. We showed that fluoride exposure reduced the expression of synaptic protein synaptophysin (SYN) and impaired learning and memory functions, whereas CS counteracted these alterations, suggesting its protective effect against fluoride-induced cognitive deficits. Further studies revealed disruption of the Erk1/2/MMP-2/MMP-9 signaling pathway both in vivo and in vitro, manifested by increased total Erk1/2, Erk1/2 phosphorylation and MMP-9 expression, along with decreased MMP-2 levels. Importantly, treatment of SH-SY5Y cells with PD98059 or CS attenuated fluoride-induced effects, indicating a regulatory role of CS in the Erk1/2/MMP-9 signaling pathway. However, MMP-2 was not implicated in this process. These data demonstrate the neuroprotective effects of CS and highlight its potential for protecting against fluoride-induced neurotoxicity and synaptic impairment.

**Supplementary Information:**

The online version contains supplementary material available at 10.1038/s41598-025-14631-7.

## Introduction

Fluoride, a naturally occurring mineral in water and various foods, is essential for maintaining dental health. However, excessive intake can cause dental and skeletal fluorosis. Critically, elevated fluoride levels may adversely affect the central nervous system (CNS). Studies indicate that high fluoride exposure during critical brain development periods induces neurocognitive deficits, including learning impairment^1^memory deficits^2^attention deficits^3^and behavioral issues^4^. In severe cases, it may cause intellectual disability and long-term neurological disorders^5^. Epidemiological investigations^6–8^ report significantly reduced intelligence quotient (IQ) among children in endemic fluorosis regions. Additionally, extensive in vivo and in vitro studies^9–11^ demonstrate decreased cognitive function and impaired synaptic plasticity. Further evidence reveals that fluoride exposure reduces hippocampal dendrite density and synaptic vesicle levels^12^.

Synaptic plasticity refers to the capacity of synapses to undergo structural and functional modifications in response to stimulation. These modifications include changes in chemical and physical properties of presynaptic and postsynaptic components, as well as alterations in neurotransmitter types and concentrations within the synaptic cleft^13^. This process plays a crucial role in learning and memory. During learning, synaptic plasticity primarily manifests as changes in synaptic strength: when neurons receive external stimuli, the presynaptic membrane releases neurotransmitters that bind to postsynaptic receptors, triggering electrochemical cascades^14^. Repetitive stimulation enhances synaptic transmission efficiency, establishing stable synaptic connections^15^. For memory formation, synapses integrate prior experiences through re-stimulation of existing connections, optimizing information transfer^16^. Conversely, aberrant synaptic plasticity impairs learning and memory. Recent studies demonstrate that fluoride triggers mitochondrial oxidative stress and dysfunction, causing synaptic impairment in the mouse hippocampus^17^. Additionally, fluoride disrupts cytoskeletal assembly and synapse formation^18,19^and mediates synaptic damage that reduces hippocampal plasticity during development^20^. Collectively, these studies confirm that fluoride decreases synaptic plasticity in rodent brains. Synaptophysin (SYN), a presynaptic vesicle protein^21^serves as a reliable marker for quantifying synaptic vesicles and assessing neurotransmission capacity. Its expression accurately reflects synaptic structural and functional integrity^22^. Thus, fluoride-induced cognitive deficits primarily stem from impaired synaptic plasticity, highlighting the need to investigate synapse-related pathways and develop interventions to restore plasticity.

Extracellular signal-regulated kinase 1/2 (Erk1/2) belongs to the mitogen-activated protein kinase family and plays a pivotal role in cellular signaling, physiological functions, and pathological mechanisms^23^. Studies have shown that Erk1/2 regulates cellular processes including growth, proliferation, and survival^24^. Consequently, abnormal Erk1/2 activation is linked to numerous disorders, particularly those affecting the CNS. Accumulating evidence indicates that fluoride activates the Erk1/2 signaling pathway in brain tissue. Our previous studies^25^ demonstrated that fluoride increases Erk1/2 phosphorylation levels in rat brains, compromising learning and memory. Moreover, Erk1/2 pathway activation impairs synaptic plasticity and subsequently alters cognitive functions^26^. Fluoride induces synaptic plasticity deficits in rats via the Erk1/2 pathway, which can be attenuated by the inhibitor PD98059^27^, thereby protecting synaptic integrity and cognitive functions.

Matrix metalloproteinase-2 (MMP-2) and matrix metalloproteinase-9 (MMP-9), members of the collagenase subclass, are crucial components of the MMP family^28^. They regulate extracellular matrix (ECM) degradation and remodeling^29^thereby influencing physiological processes including cell migration, tissue remodeling, and angiogenesis. However, aberrant MMP-2/MMP-9 activity disrupts synaptic structure and function through ECM degradation, neuronal inflammation^30^oxidative stress^31^and impaired neuronal signaling^32^. Notably, Erk1/2 signaling activation upregulates MMP-9 expression, promoting ECM degradation that compromises synaptic stability and ultimately impairs learning and memory^33,34^. Thus, modulating MMP-2/MMP-9 activity could preserve synaptic plasticity and cognitive function. Importantly, MMP-2 and MMP-9 exhibit region-specific expression in fluorotic rats^35^suggesting that fluoride induces differential MMP expression via Erk1/2 signaling, altering cognitive function. Nevertheless, no studies have directly investigated MMP-mediated synaptic plasticity in fluorosis-induced cognitive impairment.

Chondroitin sulfate (CS), a naturally occurring glycosaminoglycan, is abundant in human cartilage and the central nervous system. As a vital cartilage matrix component, CS promotes chondrocyte metabolism and proliferation, ensuring normal cartilage growth and repair. Recent studies^36^ demonstrate that CS alleviates neuroinflammation and oxidative stress, protecting neurons from damage and death. Furthermore, CS promotes neuronal growth, differentiation, and synaptic transmission^37^. Studies^38,39^ show that CS regulates cellular signaling and differentiation by activating Erk1/2 pathways in peripheral systems; however, evidence in the CNS remains limited. Notably, CS inhibits MMP-2 and MMP-9 expression in osteoarthritis^40^. Although CS exhibits multi-system protective effects, its specific neuroprotective mechanisms—particularly regarding synaptic plasticity and cognitive functions—are poorly understood.

Thus, given CS’s protective effects on synaptic plasticity and the involvement of Erk1/2-MMP-2/MMP-9 signaling in plasticity regulation, we hypothesize that CS protects against fluoride-induced cognitive deficits via the Erk1/2-MMP-2/MMP-9 pathway.

## Results

### CS mildly attenuates cognitive decline and ultrastructural damage in fluorotic rats

**Fig. 1:**
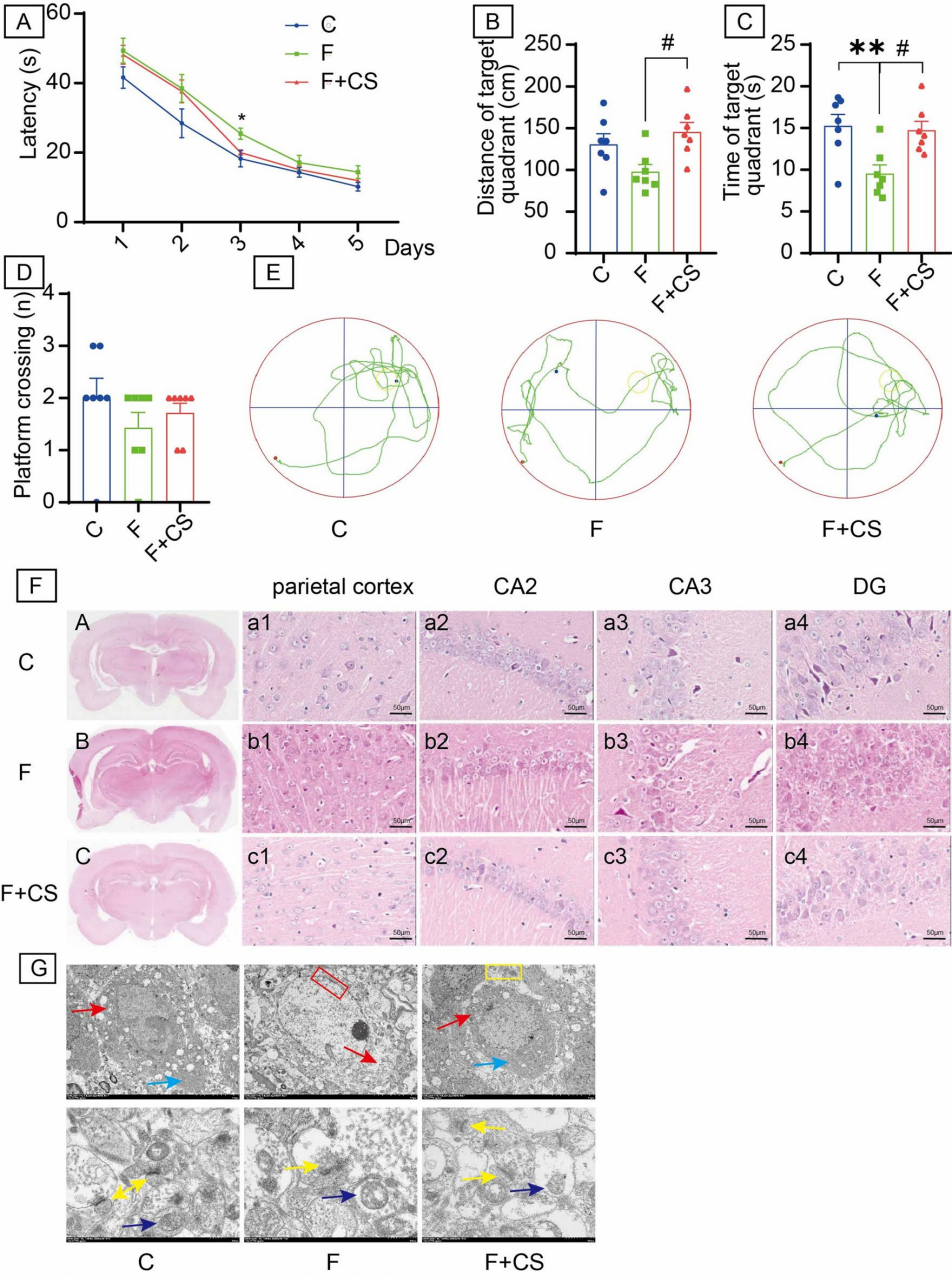
Effects of CS on cognitive (learning and memory), histology and ultrastructure of rats with fluorosis. (**A**) The escape latency during the place navigation test. Compared with the fluoride group, the escape latency of control group was observed a significant decrease on the third day’ place navigation test. (**B**) Distance spent in the target quadrant. (**C**) Time spent in the target quadrant. (**D**) Number of platform crossings. (**E**) Tracks of spatial probe test. (**F**) The pathological morphology of brain tissues was observed by HE staining. The representative images labeled as a1-c1, a2-c2, a3-c3, and a4-c4 correspond to the parietal cortex, CA2 region, CA3 region, and dentate gyrus, respectively. Bar = 50 μm. (**G**) Ultrastructural observation of the hippocampus. Light blue arrows: Endoplasmic reticulum. Red arrows: Golgi complex. Red box: chromatin condensation and margination. Yellow box: The endocytosis vesicles. Yellow arrows: The structure of synapse. Dark blue arrows: mitochondrion. Bar = 5 μm (top panel), Bar = 500 nm (bottom panel). Data are shown as the mean ± SEM; *n* = 6–7 for each group. *P < 0.05 and **P < 0.01 with the control group. ^#^*P* < 0.05 with the Fluorie group.

Spatial learning and memory were assessed using the MWM. During place navigation, mean escape latency decreased progressively; however, on day 3, the fluoride group exhibited significantly longer latencies than controls (Fig. 1A). CS administration did not reverse fluoride-induced deficits. Fluoride-exposed rats showed non-significant increases in distance traveled in the target quadrant (*P* > 0.05, Fig. 1B) and platform crossings (*P* > 0.05, Fig. 1C), but spent significantly more time in the target quadrant (*P* < 0.05, Fig. 1D). CS treatment reversed these fluoride-induced changes. Representative swim paths (day 6) demonstrated shorter target quadrant distance and fewer platform crossings in the fluoride group versus controls, while the F + CS group showed increased target quadrant distance (Fig. 1E). Collectively, these results indicate that CS counteracts learning and memory impairments in fluorotic rats.

HE staining revealed disorganized neurons with eosinophilic changes but intact nuclei in fluoride-exposed rats (Fig. 1F), unaltered by CS treatment. TEM analysis identified severe hippocampal damage in fluorotic rats, including: Cytoplasmic alterations: mitochondrial swelling, cristae rupture, dilated endoplasmic reticulum; Nuclear abnormalities: membrane shrinkage, fragmentation, displacement, and chromatin condensation; Synaptic defects: reduced postsynaptic density and blurred synaptic clefts. CS treatment ameliorated these ultrastructural alterations (Fig. 1G), partially preserving postsynaptic membrane integrity and synaptic space clarity.

### CS mitigates fluoride-induced synaptophysin reduction

**Fig. 2:**
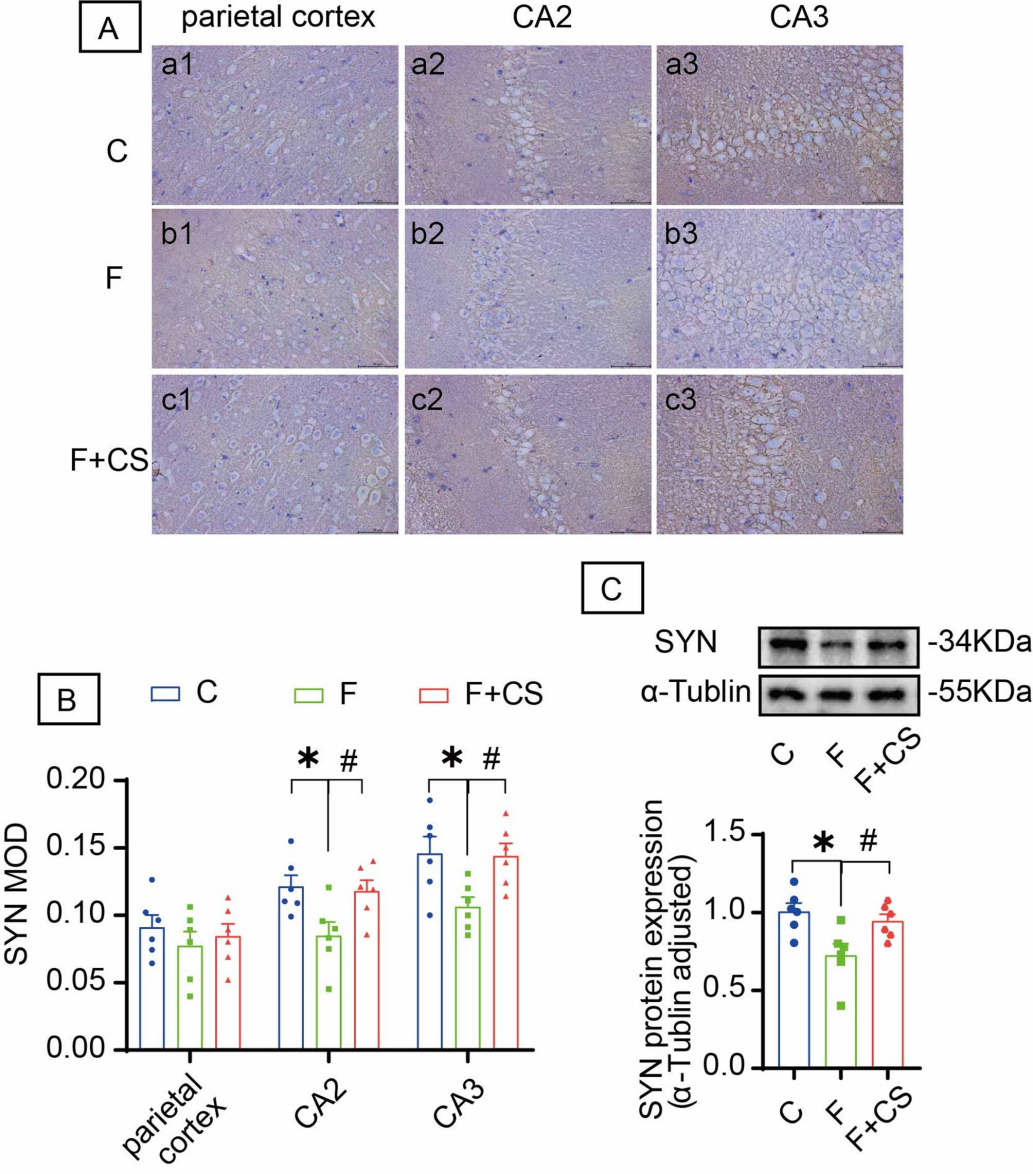
Impact of CS on SYN in various brain regions. (**A**) The protein expression level of SYN was detected by immunohistochemistry staining. The representative images labeled as a1-c1, a2-c2 and a3-c3 correspond to the parietal cortex, the CA2 region and the CA3 region, respectively. Bar = 50 μm. (**B**) Mean optical density values of SYN in the parietal cortex, the CA2 region and the CA3 regions. (**C**) The protein level of SYN was examined using Western blot analysis. The samples derive from the same experiment and that blots were processed in parallel. Original blots are presented in Supplementary Fig. 1. Data are shown as the mean ± SEM; *n* = 6 for each group. *P < 0.05 with the control group. ^#^*P* < 0.05 with the Fluorie group.

IHC revealed predominant SYN expression on plasma membranes (Fig. 2A). Fluoride exposure significantly reduced SYN protein levels in hippocampal CA2 and CA3 regions. CS treatment reversed this reduction (Fig. 2B). Western blot analysis confirmed these findings (Fig. 2C). Collectively, these results demonstrate that fluoride induces synaptic damage, while CS exerts neuroprotective effects by preserving synaptic integrity.

### CS counteracts fluoride-induced phospho-Erk1/2 upregulation

**Fig. 3:**
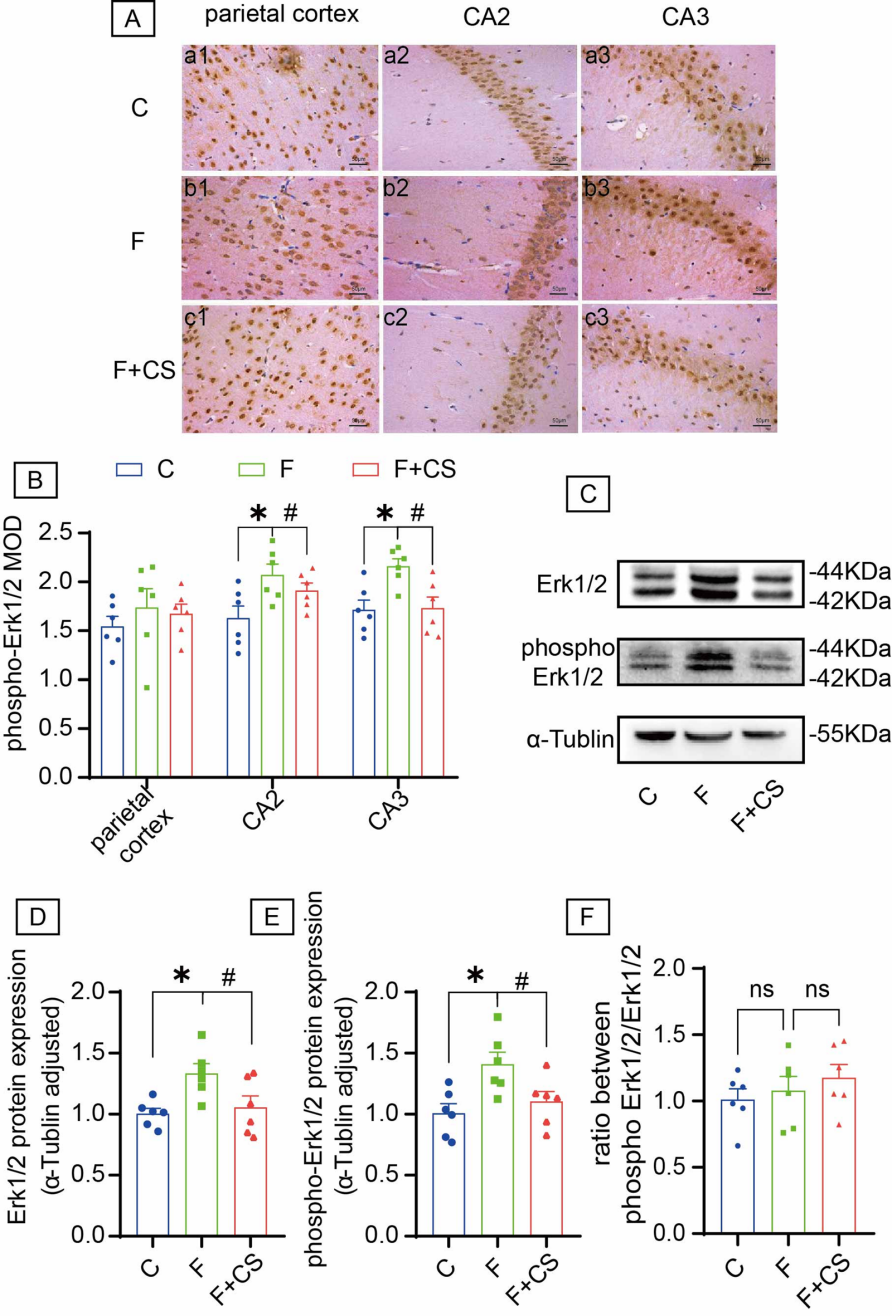
Effects of CS on Erk1/2 and phospho-Erk1/2 in the brain of rats with fluorosis. (**A**) Immunohistochemistry analysis was utilized to assess the expression levels of phospho-Erk1/2 protein. The representative images labeled as a1-c1, a2-c2, and a3-c3 correspond to the parietal cortex, the CA2 region, and the CA3 region, respectively. Bar = 50 μm. (**B**) Mean optical density values of phospho-Erk1/2 in the parietal cortex, the CA2 region and the CA3 regions. (**C**) Representative images of Western blot for Erk1/2 and phospho-Erk1/2 in brain. Quantitative analyses of Erk1/2 (**D**), phospho-Erk1/2 (**E**) protein and the ratio of phosphorylated Erk1/2 to total Erk1/2 expression levels (**F**) normalized to the internal control α-Tublin. The samples derive from the same experiment and that blots were processed in parallel. Original blots are presented in Supplementary Fig. 2. Data are shown as the mean ± SEM; *n* = 6 for each group. *P < 0.05 with the control group. ^#^*P* < 0.05 with the Fluorie group. ^ns^*P* > 0.05 .

IHC revealed predominant cytoplasmic localization of phospho-Erk1/2 (Fig. 3A). Fluoride exposure significantly increased phospho-Erk1/2 protein levels in hippocampal CA2 and CA3 regions. Notably, CS treatment reduced phospho-Erk1/2 levels in CA3 compared to fluorotic rats (Fig. 3B). Western blot further confirmed significant elevations in both total Erk1/2 and phospho-Erk1/2, and CS reversed these fluoride-induced changes, as evidenced by decreased protein levels in CS-treated groups (Fig. 3C-E). Moreover, the ratio of phosphorylated Erk1/2 to total Erk1/2 showed no significant alteration when comparing control and CS-treated rats with the fluoride-exposed group (Fig. 3F). These findings indicate that Erk1/2 signaling mediates fluoride neurotoxicity.

### CS suppresses fluoride-triggered MMP-9 overexpression

**Fig. 4:**
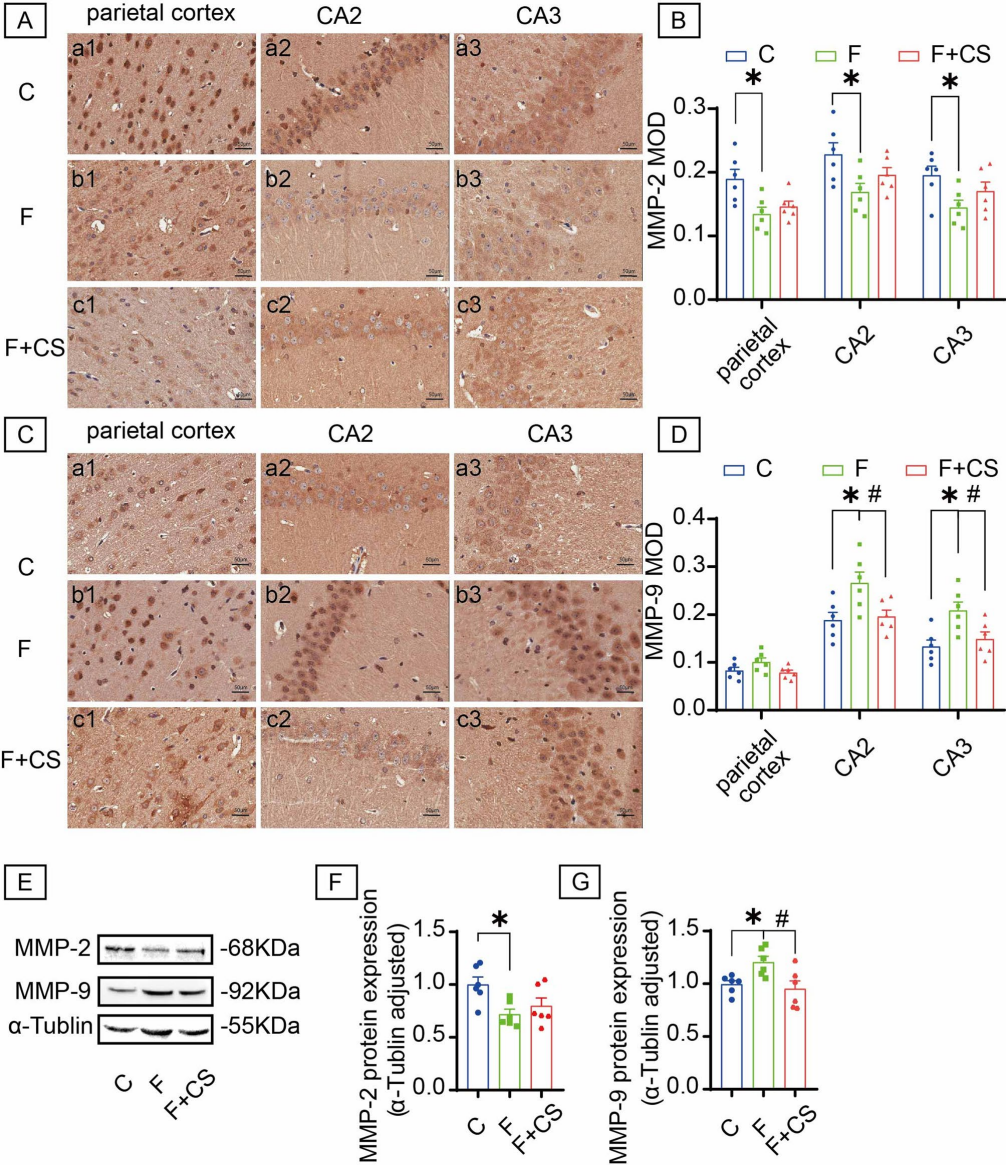
Effects of CS on MMP-2 and MMP-9 in the brain of rats with fluorosis. The expression levels of MMP-2 protein (**A**) and MMP-9 protein (**C**) was detected by immunohistochemistry analysis. The representative samples labeled as a1-c1, a2-c2 and a3-c3 correspond to the parietal cortex, the CA2 region and the CA3 region, respectively. Bar = 50 μm. Mean optical density values of MMP-2 (**B**) and MMP-9 (**D**). Representative images of Western blot for MMP-2 protein and MMP-9 protein in brain (**E**). Quantitative analyses of MMP-2 (**F**) and MMP-9 (**G**) protein expression levels normalized to the internal control α-Tublin. The samples derive from the same experiment and that blots were processed in parallel. Original blots are presented in Supplementary Fig. 3. Data are shown as the mean ± SEM; *n* = 6 for each group. * P < 0.05 with the control group. ^#^*P* < 0.05 with the Fluorie group.

IHC revealed cytoplasmic localization of MMP-2 and MMP-9 (Fig. 4A, C). Fluoride exposure significantly decreased MMP-2 and increased MMP-9 protein levels. Specifically, MMP-2 reduction occurred in hippocampal CA2, CA3, and parietal cortex, unaffected by CS treatment (Fig. 4B). Conversely, MMP-9 elevation in CA2/CA3 was significantly attenuated by CS (Fig. 4D). Western blot confirmed that fluoride-exposed rats exhibited lower MMP-2 (Fig. 4E, F) and higher MMP-9 (Fig. 4E, G) versus controls, with CS selectively reducing MMP-9 but not MMP-2 levels. These findings demonstrate that CS protects against fluoride-induced MMP-9 upregulation.

### Inhibition of Erk1/2-MMP-2/MMP-9 signaling reverses fluoride-induced synaptic damage in SH-SY5Y cells

Given the established role of Erk1/2 signaling in fluoride neurotoxicity, we investigated whether fluoride regulates MMP-2/MMP-9 through this pathway to induce synaptic damage. SH-SY5Y cells were treated with PD98059 (an Erk1/2 inhibitor) alongside fluoride exposure.

**Fig. 5:**
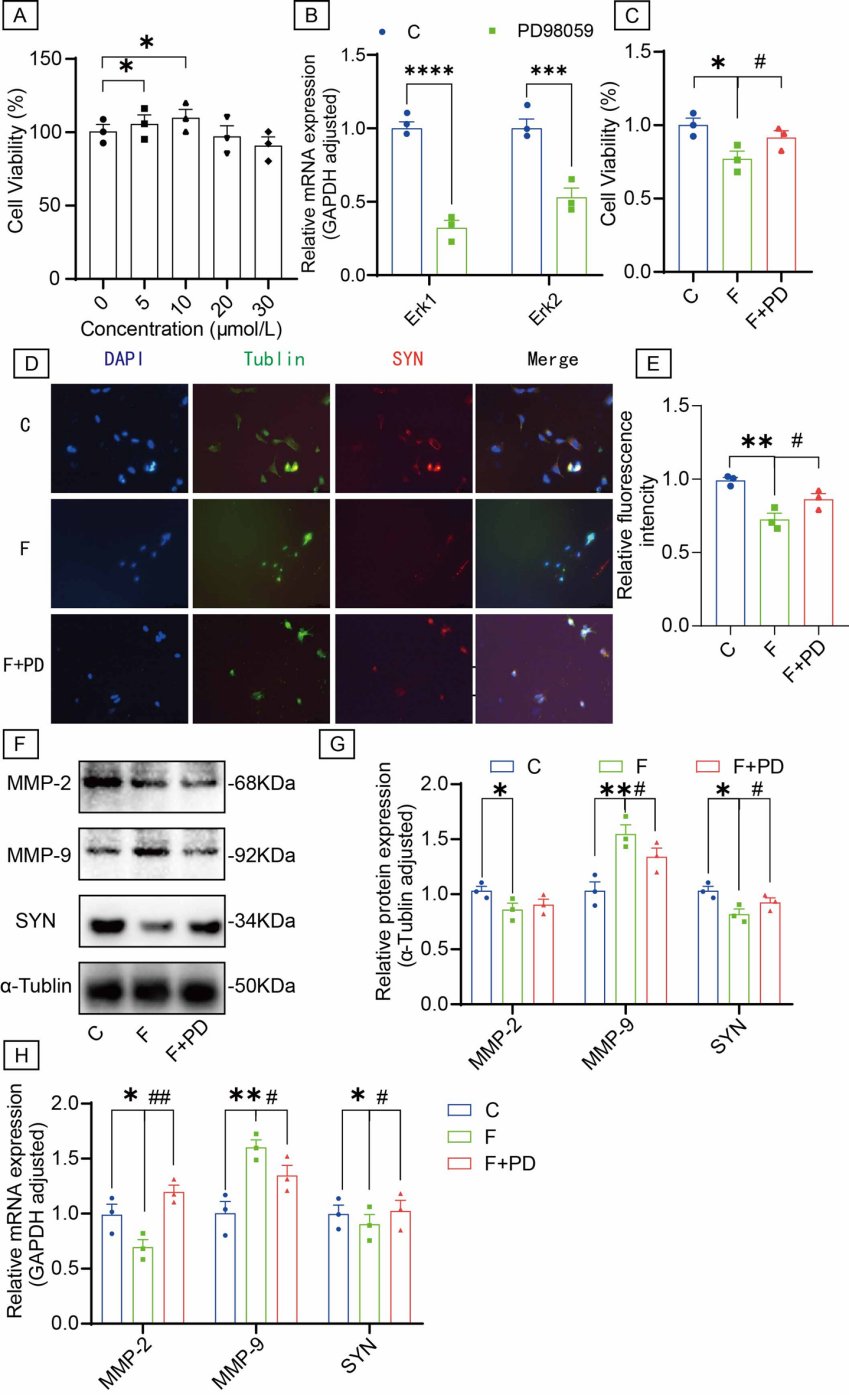
The Erk1/2/MMP-2/MMP-9 signaling pathway inhibition reversed synaptic changes induced by fluoride in SH-SY5Y cells. (**A**) CCK-8 assay was detected to cell viability of SH-SY5Y cells treated with the different concentrations of PD98059 (0, 5, 10, 20, 30 µmol/L) for 48 h. (**B**) Quantification of PD98059-mediated suppression of Erk1/2 gene expression by RT-qPCR. (**C**) The cell viability of SH-SY5Y cells among three groups rats after PD98059 (10 µmol/L) treatment for 48 h. (**D**) The immunofluorescence staining and (**E**) quantification analysis of SYN protein in SH-SY5Y cells. (**F**) The protein levels of MMP-2, MMP-9 and SYN were examined by Western blot in SH-SY5Y cells. (**G**) Quantitative analyses of MMP-2, MMP-9 and SYN protein expression levels normalized to the internal control α-Tublin. (**H**) RT-qPCR was used to measure the expression levels MMP-2, MMP-9 and SYN gene in SH-SY5Y cells. The samples derive from the same experiment and that blots were processed in parallel. Original blots are presented in Supplementary Fig. 4. Data are shown as the mean ± SEM; *n* = 3 for each group. *P < 0.05, **P < 0.01, ***P < 0.001 and ****P < 0.0001 with the control group. ^#^*P* < 0.05 and ^##^*P* < 0.01 with the Fluorie group.

The optimal PD98059 concentration was determined as 10 µmol/L (48 h treatment) (Fig. 5A), achieving inhibition efficiencies of 32.29% for Erk1 and 53.07% for Erk2 at this dose (Fig. 5B). Fluoride significantly reduced cell viability (Fig. 5C) and downregulated synaptophysin (SYN) protein expression, effects that were effectively rescued by PD98059 co-treatment (Figs. 5D-E). Furthermore, fluoride downregulated MMP-2 and SYN mRNA/protein levels while upregulating MMP-9 mRNA/protein expression (Figs. 5F-H), alterations that were reversed by PD98059 (with the exception of MMP-2 mRNA). These results demonstrate that fluoride induces synaptic damage through Erk1/2-mediated regulation of MMP-2/MMP-9.

### CS restores synaptic function in fluoride-treated SH-SY5Y cells by suppressing Erk1/2-MMP-9 signaling

**Fig. 6:**
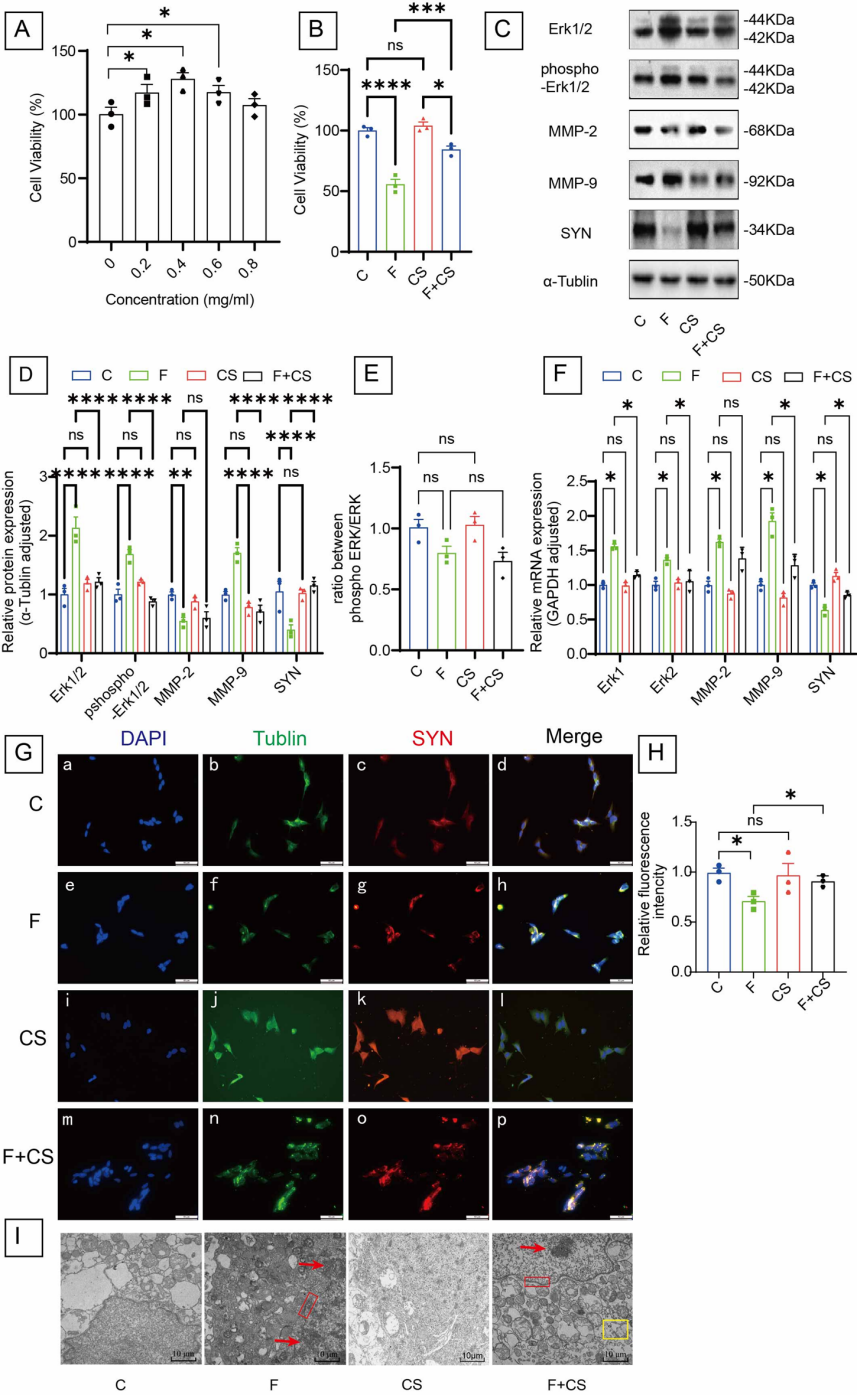
CS suppressed synaptic damage induced by fluoride in SH-SY5Y cells. (**A**) Cell viability of SH-SY5Y cells treated with the CS of different concentrations (0.2, 0.4, 0.6, 0.8 mg/L) for 48 h were detected by CCK-8 assay. (**B**) CCK-8 was used to examine the cell viability of SH-SY5Y cells after CS (0.4 mg/ml) treatment for 48 h. (**C**) The Western blot and (**D**) quantification analysis of Erk1/2, phospho-Erk1/2, MMP-2, MMP-9 and SYN in SH-SY5Y cells. (**E**) The ratio of phosphorylated Erk1/2 to total Erk1/2 expression levels. (**F**) RT-qPCR analysis of Erk1/2, MMP-2, MMP-9 and SYN gene in SH-SY5Y cells. (**G**) The immunofluorescence staining and (**H**) quantification analysis of SYN in SH-SY5Y cells. (**I**) Ultrastructural observation of the SH-SY5Y cells. Red arrows: chromatin condensation and margination. Red box: integrity of the nuclear membrane disappeared. Yellow box: The endocytosis vesicles. Bar = 10 μm. The samples derive from the same experiment and that blots were processed in parallel. Original blots are presented in Supplementary Fig. 5. Data are shown as the mean ± SEM; *n* = 3 for each group. * P < 0.05, ** P < 0.01, *** P < 0.001 and **** P < 0.0001. ^ns^*P >* 0.05.

**Fig. 7:**
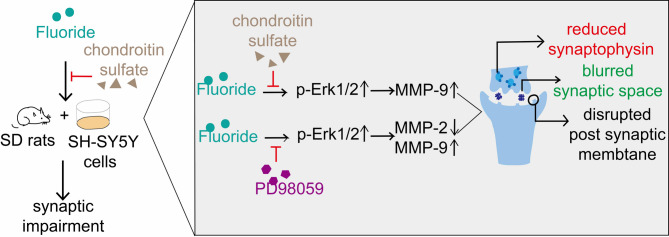
Diagram depicting the mechanism of CS protected against synaptic impairment caused by fluorosis through the Erk1/2-MMP-9 signaling pathway.

Building upon the in vivo evidence for the neuroprotective effects of CS, we investigated its underlying mechanism in SH-SY5Y cells. A 48 h incubation experiment identified 0.4 mg/ml as the optimal CS concentration (Fig. 6A). Compared to the fluoride-treated group, CS significantly enhanced cell viability (Fig. 6B), and the control group treated with CS alone did not exhibit cytotoxicity. At both the protein (Fig. 6C-D) and mRNA levels (Fig. 6F), CS effectively suppressed Erk1/2 and MMP-9 expression while simultaneously upregulating SYN expression. Additionally, the p-ERK/tERK ratio remained unchanged (Fig. 6E). Immunofluorescence experiments further confirmed that CS reversed the fluoride-induced reduction in synaptophysin protein (Fig. 6G-H), consistent with the qPCR and Western blot data (Fig. 7).

Notably, CS formed peri-cellular aggregates visible by immunofluorescence (Fig. 6G). TEM revealed enhanced endocytosis in CS-treated versus control/fluoride groups (Fig. 6I), suggesting internalization may mediate neuroprotection. Whereas fluoride induces synaptic damage via the Erk1/2-MMP-2/MMP-9 axis, CS confers protection by inhibiting this pathway.

This study demonstrates that fluoride triggers synaptic damage in SH-SY5Y cells primarily through the Erk1/2-MMP-9 cascade, with CS exerting protective effects via targeted suppression of this signaling axis.

## Discussion

Fluoride is an essential trace element for the human body; however, excessive fluoride intake can lead to fluorosis, causing toxicological damage to multiple organs, including the central nervous system^1^. Notably, fluoride can cross the blood-brain barrier, accumulate in brain tissue, and affect brain function. Studies indicate that excessive fluoride exposure can trigger disturbances in cerebral calcium metabolism, oxidative stress^10^neuroinflammation^12^and crucially, abnormalities in synaptic plasticity^13^. Given that synaptic plasticity serves as the neural foundation for learning and memory, its susceptibility to fluoride-induced damage suggests this may be a key mechanism underlying fluoride’s impairment of cognitive function. Therefore, in-depth investigation into the mechanisms by which fluoride affects synaptic plasticity holds significant scientific importance.

CS, a glycosaminoglycan with diverse bioactivities, shows promising potential in the field of neuroprotection. Research indicates that CS can repair neural cells, promote neuronal growth, delay neuronal death, counteract neuronal damage, and ameliorate cognitive impairment (particularly in learning and memory) as well as depressive symptoms^41^. These beneficial effects are closely associated with its pharmacological activities, including antioxidant and anti-inflammatory properties^42^and crucially, the modulation of synaptic plasticity^43^. Furthermore, CS and its proteoglycans play a significant role in maintaining neuronal homeostasis by regulating neuronal plasticity and interacting with key proteins^44^. Based on this evidence, the present study aims to investigate whether and how CS antagonizes fluoride-induced impairments in synaptic plasticity and learning and memory capabilities.

To validate the neuroprotective effects of CS, we initially established a rat model of fluorosis and administered CS, subsequently evaluating its impact on learning and memory capabilities^45^. The Morris water maze test revealed that: During the navigation trials, the escape latency was significantly prolonged in the fluorosis group compared to the control group, consistent with the findings reported by Liu et al.^25^indicating impaired spatial learning ability due to fluoride exposure. In the spatial probe test, although the number of platform crossings did not show a statistically significant difference, both the time spent in the target quadrant and the distance traveled within it were significantly reduced. Collectively, these results suggest that fluoride exposure likely impairs spatial memory consolidation. The discrepancy with some previous rat studies^20^ might be attributable to variations in drug dosage, timing of administration, or experimental protocols. Importantly, CS intervention significantly ameliorated the performance of fluorotic rats in the water maze test, suggesting its protective effect against fluoride-induced learning and memory impairments.

Given that synaptic plasticity is a core mechanism underlying learning and memory function, we further examined changes in neuronal morphology and synaptic structure within the brain tissue. HE staining revealed disorganized neuronal arrangement accompanied by eosinophilic changes in fluorotic rats, consistent with previous findings^3^; however, morphological improvement was not evident following CS treatment. Nevertheless, TEM results uncovered more subtle alterations: Fluorotic rats exhibited significant degenerative alterations in hippocampal neurons, manifested as a reduction in postsynaptic density and blurring of the synaptic cleft. This observation aligns with reported phenomena of organelle damage and loss^46^. Critically, these ultrastructural impairments were significantly ameliorated by CS intervention, indicating a protective effect of CS on the postsynaptic membrane structure and the widened synaptic cleft.

To delve deeper into the intrinsic mechanisms underlying the neuroprotective effects of CS, we focused on signaling pathways potentially implicated in both fluoride neurotoxicity and the regulation of synaptic plasticity. Multiple mechanisms—including oxidative stress, endoplasmic reticulum stress, inflammatory responses, and activation of specific signaling pathways—are thought to contribute to the toxic effects of fluoride^1^. Among these, the Erk1/2 signaling pathway plays a critical role in brain development and functional activities, encompassing synaptic plasticity and learning and memory. Previous studies have demonstrated that inhibition of Erk1/2 pathway activation leads to decreased SYN expression and impairments in learning and memory^47–49^. Consistent with our preliminary study^25^ and confirmed in the present experiment, fluorosis induced aberrant activation of the Erk1/2 signaling pathway in the hippocampal CA2 and CA3 regions of rats, which was closely associated with learning and memory dysfunction.

Matrix metalloproteinases MMP-2 and MMP-9 are key regulatory factors of the ECM, playing significant roles in synaptic plasticity and learning and memory^50,51^. MMP-9, in particular, exhibits a central function in regulating synaptic plasticity within the prefrontal cortex, amygdala, and hippocampus^34,52^ where enhanced MMP-9 activity can promote long-term potentiation and facilitate learning and memory. However, its role is complex and context-dependent: While MMP-9 deficiency is associated with learning and memory decline, clinical studies have also observed significantly elevated MMP-9 levels in the brain tissue, cerebrospinal fluid, and plasma of patients with cognitive impairment, such as Alzheimer’s disease^53–55^. This elevation may be linked to neuroinflammatory responses and pathological damage processes. To explore another potential mechanism through which CS influences synaptic plasticity, we investigated its effects on MMP-2 and MMP-9 activity in the brain tissue of fluorotic rats. Our experiments revealed a differential response: Fluorosis induced a significant decrease in MMP-2 activity alongside a marked increase in MMP-9 activity within the CA2 and CA3 hippocampal regions. Strikingly, CS intervention significantly reduced this abnormally elevated MMP-9 activity, while exerting a minimal effect on MMP-2 activity. This finding, particularly the observed MMP-9 increase, differs from some previous studies (especially those focusing on cortical regions)^35^. This discrepancy may stem from the complex interactions between fluoride and the intra/extracellular environment, reflecting the multifaceted nature of biochemical regulation. Given the critical, albeit complex, role of MMP-9 in synaptic plasticity, we propose that the significant regulation of MMP-9 activity by CS represents another important mechanism contributing to its amelioration of fluoride-induced synaptic damage.

In summary, fluoride impairs neuron-synapse crosstalk through Erk1/2-MMP-9 signaling activation. CS exerts neuroprotective effects through the following mechanisms: inhibiting total Erk1/2 and its hyperphosphorylation to block upstream signal transduction, selectively suppressing excessive MMP-9 activation in hippocampal microregions, and rescuing the expression of synaptic proteins (particularly synaptophysin) to restore structural plasticity. This coordinated intervention normalizes extracellular matrix dynamics critical for neural network function. Future investigations should delineate CS-mediated astrocytic ECM remodeling and its potentiation of neurite outgrowth in chronic fluorosis, particularly examining CSPG-mediated neurotrophic crosstalk in the tripartite synapse microenvironment.

## Materials and methods

### Materials

CS was purchased from ChemFaces (Changde, China). PD98059 (an Erk1/2 inhibitor) was purchased from MedChemExpress (Shanghai, China). Dulbecco’s modified eagle’s medium (DMEM) and fetal bovine serum were purchased from GIBCO (Carlsbad, California, USA). Antibodies used in Western blot analysis and immunohistochemical included those against Erk1/2 (11257-1-AP), phospho-Erk1/2 (28733-1-AP), SYN (67864-Ig) and Tublin beta (10094-1-AP) antibodies were obtained from Proteintech Group, Inc; MMP2 (#AF5228) and MMP9 (#AF5330)antibodies were obtained from Affinity Biosciences.

### Experimental animals

Thirty SD rats (half males and half females, and weighting 90–120 g) were purchased from the Experimental Animal Center in Guizhou, China, and ethical permission for these experiments was obtained from the regional ethical committee for animal studies in Guizhou (SYXK(QIAN)2018-0001), conducted according to the INSERM animal care and are in compliance with the DIRECTIVE 2010/63/EU of the European Parliament. The humidity ranged from 30 to 55% and temperature remained between 22 and 25 °C. The rats were acclimatized for one week in a housing facility before treatment.

The rats were randomly divided into 3 groups with 10 rats in each group. The control group was supplied with drinking water containing fluoride less than 0.5 mg/L, fluoride exposed group was supplied with drinking water containing 10 mg/L fluoride and CS group was intraperitoneally injected with 0.66 mg/kg CS for 5 days after supplied with drinking water containing 10 mg/L fluoride for 90 days. At the end of the experiment, the rats were deprived of food for a 12-hour period preceding the surgical procedure. The rats were then positioned within an anesthesia induction chamber, where they were exposed to 5% sevoflurane to induce anesthesia. As soon as the rats’ limbs relaxed, they were moved to a chamber with a high concentration of carbon dioxide for euthanasia. Following roughly five minutes of exposure to carbon dioxide, the rats succumbed to unconsciousness and subsequently passed away. The rats were perfused with PBS and the brains were fixed with 10% neutral formaldehyde.

### Cell culture and treatment

SH-SY5Y cells, a human neuroblastoma cell line purchased from the German Collection of Microorganisms and Cell Culture (Germany), were cultured in high glucose DMEM medium (Gibco, USA) supplemented with heat-inactivated fetal bovine serum (Gibco, USA) and 25 units of penicillin-streptomycin/ml (Solarbio, China) and cultured in 5%CO_2_ at 37 °C incubator. The cells were divided into control group, fluoride group, inhibitor + fluoride group, CS group, CS + fluoride group; in fluoride group, the SH-SY5Y cells were incubated in medium with fluoride ion 4 mmol/L for 48 h; in inhibitor + fluoride group, the cells were pretreated with inhibitor PD98059 10 µmol/L for one h before incubated with fluoride ion; in CS group, the SH-SY5Y cells were incubated in medium with CS 0.4 mg/ml for 48 h; in CS + fluoride group, the cells were incubated with CS 0.4 mg/ml and fluoride ion 4 mmol/L for 48 h.

### Morris water maze (MWM) test

Spatial learning and memory were assessed using the Morris water maze (MWM) test following established protocols^56^. The apparatus consisted of a circular metal pool (diameter: 180 cm) filled with opaque tap water (dark ink added). A stainless-steel escape platform (diameter: 9 cm) was submerged 0.5 cm below the water surface. Rats underwent 5 consecutive days of learning trials (4 trials/day) with 5–7 min inter-trial intervals. Movement was tracked using VisuTrack Rodent Behavior Video Analysis Software (V2.0) (http://www.softmaze.com). Escape latency (time to locate the hidden platform) was recorded during navigation trials. Upon platform discovery, rats remained on it for 2 s. Animals failing to find the platform within 60 s were guided to it and permitted to remain for 2 s, with latency recorded as 60 s. Daily trial latencies were averaged for analysis. On day 6, the platform was removed, and first crossing time at the original platform location was recorded. All tests occurred under subdued lighting in a sound-attenuated environment.

### Hematoxylin-eosinnt (HE) staining

Hippocampal and parietal cortex morphology was assessed using HE staining. Brains were fixed in 10% neutral buffered formalin overnight, then dehydrated through an ethanol series, cleared in xylene, embedded in paraffin, and sectioned at 3 μm thickness. Tissue sections were deparaffinized through three sequential 10-min xylene washes, then rehydrated in a graded ethanol series (100% × 2, 90% × 2, 80% × 2, 70%, 50%; 5 min per concentration). Slides were stained with hematoxylin for 2 min and eosin for 1 min, then mounted with neutral resin. Morphological analysis was conducted using an Olympus BX53 light microscope (Japan).

### Transmission electron microscopy (TEM)

Transmission electron microscopy (TEM) was employed to examine hippocampal ultrastructure following established protocols^57^. Following anesthesia, rats underwent transcardial perfusion with phosphate-buffered saline (PBS). Brains were then post-fixed in 2.5% glutaraldehyde for 2 h at 4 °C. Tissues were dehydrated through a graded acetone series (30%, 50%, 70%, 90%, 100%), embedded in EPON 812 resin, and polymerized at 60 °C for 48 h. Ultrathin section (70 nm) were prepared using a Leica UC7 ultramicrotome, double-stained with uranyl acetate (15 min) and lead citrate (5 min), and examined using a Hitachi HT7800 transmission electron microscope.

### Immunohistochemical (IHC) staining

Immunohistochemical staining was performed on hippocampal and parietal cortex sections to evaluate Erk1/2, MMP-2, MMP-9, and SYN expression according to established protocols^58^. Briefly, Sections underwent deparaffinization, rehydration, and distilled water rinsing. Antigen retrieval utilized EDTA buffer (pH 9.0) under high pressure (15 min) followed by three 5-min phosphate-buffered saline (PBS) washes. Endogenous peroxidase blocking with 3% H_2_O_2_ (30 min, RT) and non-specific binding blocking with 10% normal goat serum (30 min, 37 °C) were performed, each followed by PBS washes. Primary antibody incubations occurred overnight at 4 °C: anti-Erk1/2 (Proteintech 11257-1-AP, 1:500), anti-MMP-2 (Affinity AF5228, 1:50), anti-MMP-9 (Affinity AF5330, 1:100), and anti-SYN (Proteintech 67864-Ig, 1:500). After PBS washes, sections were incubated with biotinylated goat anti-rabbit IgG-HRP (Proteintech, 1:1000) and goat anti-mouse IgG-HRP (Proteintech, 1:1000) (30 min, 37 °C). Signal development employed diaminobenzidine (DAB, 5 min) with hematoxylin counterstaining. Five random fields per section were analyzed using an Olympus BX53 microscope, with average optical density quantified in ImageJ (v1.8.0).

### Western blot

Western blot was performed for the protein of Erk1/2, phospho-Erk1/2, MMP-2, MMP-9 and SYN following established protocols^59^. Brain tissues were homogenized in RIPA buffer using a high-speed low-temperature homogenizer, while SH-SY5Y cells were lysed in RIPA buffer (30 min, 4 °C). Protein concentrations were quantified using the bicinchoninic acid (BCA) assay. Samples underwent SDS-PAGE electrophoresis (120 V, 60 min) followed by transfer to 0.45 μm PVDF membranes (400 mA, 30 min). Membranes were blocked with 5% skim milk (60 min, RT) and incubated overnight at 4 °C with primary antibodies: anti-Erk1/2 (Proteintech 11257-1-AP; 1:2000), anti-phospho-Erk1/2 (Proteintech 28733-1-AP; 1:2000), anti-MMP-2 (Affinity AF5228; 1:5000), anti-MMP-9 (Affinity AF5330; 1:5000), and anti-SYN (Proteintech 67864-Ig; 1:8000). After three TBST washes, membranes were incubated with IRDye® secondary antibodies: goat anti-rabbit IgG (Proteintech; 1:10,000) and goat anti-mouse IgG (Proteintech; 1:10,000) (60 min, RT). Following final TBST washes, protein bands were visualized using an Odyssey CLx Imaging System.

### Cell counting kit-8 (CCK-8)

Cell viability was assessed using the Cell Counting Kit-8 (CCK-8) assay according to established protocols^60^. SH-SY5Y cells were seeded in 96-well plates and cultured for 72 h. Cells were preincubated with rat serum containing either PD98059 (0, 5, 10, 20, or 30 µmol/L) or chondroitin sulfate (CS; 0, 0.2, 0.4, 0.6, or 0.8 mg/mL) for 1 h prior to fluoride exposure, followed by 48 h incubation. Culture medium was then replaced with 10% CCK-8 solution per well, and plates were incubated at 37 °C for 4 h. Absorbance at 450 nm (OD_450_) was measured using a microplate reader (BioTek Synergy H1), with cell viability calculated relative to control groups.

### Real-time quantitative polymerase chain reaction (RT-qPCR)

RT-qPCR was performed to analyze mRNA expression of Erk1/2, MMP-2, MMP-9, and SYN in SH-SY5Y cells, performed according to the protocol established by Livak et al.^61^. Total RNA was extracted using TRIzol reagent (Invitrogen), with purity and concentration determined by NanoDrop 2000 spectrophotometry. cDNA synthesis from total RNA utilized the PrimeScript RT Reagent Kit (TaKaRa, #RR047A, Japan). Quantitative PCR reactions (10 µL volume) employed TB Green Premix Ex Taq II (TaKaRa, #RR820A, Japan) on a Bio-Rad CFX96 Real-Time PCR System. Relative mRNA expression was calculated using the 2^−ΔΔCT^ method, with gene-specific primer sequences detailed in Table 1.

**Table 1:** The primers used for RT-qPCR analysis.

Gene	Primer sequences
GAPDH	Forward 5′-cac cca. ctc ctc cac ctt tga c-3′
	Reverse 5′-gtc cac cac cct gtt gct gta g-3′
ERK 1	Forward 5′-acc tgc tca tca aca cca. cct g-3′
	Reverse 5′-gcg tag cca. cat act ccg tca g-3′
ERK 2	Forward 5′-ctg ttc cca. aat gct gac tcc aaa g-3′
	Reverse 5′-ctc gtc act cgg gtc gta ata ctg-3′
MMP-2	Forward 5′-acc tac acc aag aac ttc cgt ctg-3′
	Reverse 5′-tgc caa ggt caa tgt cag gag ag-3′
MMP-9	Forward 5′-tgg tcc tgg tgc tcc tgg tg-3′
	Reverse 5′-tgc ctg tcg gtg aga ttg gtt c-3′
SYN	Forward 5′-ctg ctg ctg gcg gac atg g-3′
	Reverse 5′-aag gcg aag atg gcg aag acc-3′

### Immunofluorescence (IF)

IF was performed to analyze SYN expression according to established protocols^62^. SH-SY5Y cells were fixed with 4% paraformaldehyde (PFA) for 20 min at RT, followed by three PBS washes. Cells were permeabilized with 0.5% Triton X-100 in PBS for 10 min at RT and washed thrice with PBS. Non-specific binding was blocked with 10% bovine serum albumin (BSA) for 30 min at 37 °C. Samples were incubated overnight at 4 °C with primary antibodies: anti-SYN (Proteintech 67864-Ig, 1:500) and anti-β-tubulin (Proteintech 10094-1-AP, 1:1000). After PBS washes, cells were incubated with fluorophore-conjugated secondary antibodies (Alexa Fluor 488 goat anti-rabbit and Alexa Fluor 594 goat anti-mouse, 1:1000) for 30 min at 37 °C. Nuclei were counterstained with 4’,6-diamidino-2-phenylindole (DAPI) for 5 min. Imaging was performed using an Olympus IX83 fluorescence microscope.

### Statistical analysis

Accurate data interpretation was achieved through the utilization of GraphPad Prism 8.0 software alongside SPSS statistics 26 for the conducted analyses. The differences among groups were presented as mean ± SEM. To evaluate statistical disparities among more than two groups, the one-way ANOVA was utilized. Statistical significance was determined when the p-value was less than 0.05.

## Supplementary Information

Below is the link to the electronic supplementary material.

Supplementary Material 1

## Data Availability

Data is provided within the manuscript or supplementary information files.

## Declarations

### ARRIVE guidelines statement

Our study is reported in accordance with ARRIVE guidelines.
