# Supplementary material for: Chondroitin sulfate protects against synaptic impairment caused by fluorosis through the Erk1/2-MMP-9 signaling pathway
Source: Sci Rep. 2025 Aug 13;15:29760. doi: 10.1038/s41598-025-14631-7 (PMC12350683; doi:10.1038/s41598-025-14631-7)
Supplement: Supplementary file 1 — Supplementary Material 1 [file 41598_2025_14631_MOESM1_ESM.pdf]

Chondroitin sulfate protected against synaptic impairment caused by fluorosis through the Erk1/2-MMP-9 signaling pathway

Fujun Ai # [1, 2], Shengyuan Wang # [3] , Ling Ye [4], Wen Wan [5], Xiao Zhou [1, 2], Minghai Liu [4], Kaiju Mo [1, 2], Yongheng Lu [1, 2], Na Wei [1, 2], Zhizhong Guan [1, 2, 5], Yanjie Liu * [1, 2]

[1] Department of Pathology in the Affiliated Hospital of Guizhou Medical University, Guiyang 550004, China

[2] Pathology Morphology and Molecular Laboratory of the Affiliated Hospital of Guizhou Medical University, Guiyang 550004, China

[3] Department of Pathology, Lin Yi People's Hospital, Linyi, Shandong, China

[4] Department of Neurology in the People's Hospital of Huaxi District, Guiyang 550025, China

[5] Department of Pathology, Guizhou Medical University, Guiyang 550004, Guizhou, China

[6] Key laboratory of Endemic and Ethnic Diseases of the Ministry of Education (Guizhou Medical University), Guizhou Medical University, Guiyang 550004, China

should be considered joint first author. Co-first author

*Corresponding author:

Liu Yanjie

Department of Pathology in the Affiliated Hospital, Guizhou Medical University, Guiyang 550004, China.E-mail: liuyanjie@gmc.edu.cn

Running title: Chondroitin sulfate protects brain damage in chronic fluorosis

Supplementary Figure 1

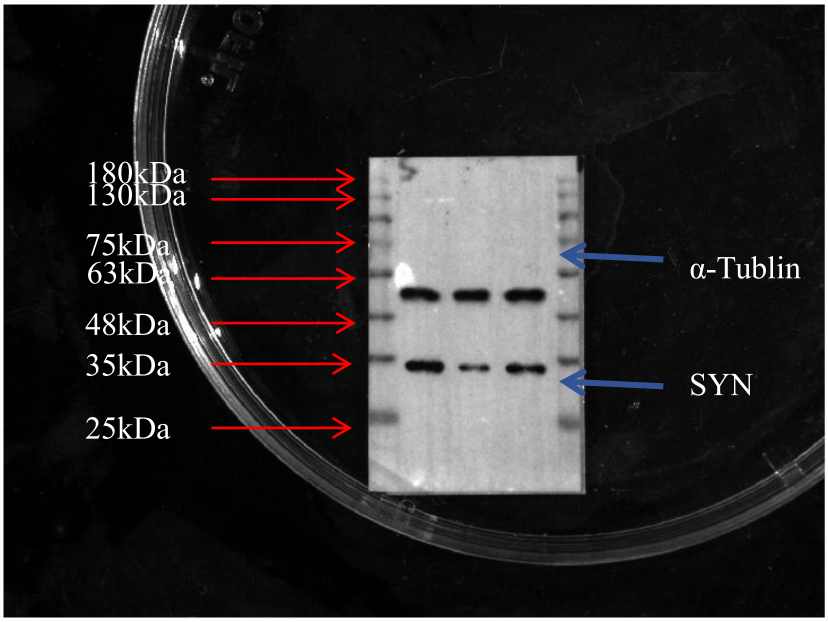

Supplementary Figure 1. The full-length blots involved in Western blot in Figure 2(α-Tublin, SYN). The full-length blots came from the same gel. These blots are used in the Figure 2C.

Supplementary Figure 2

A

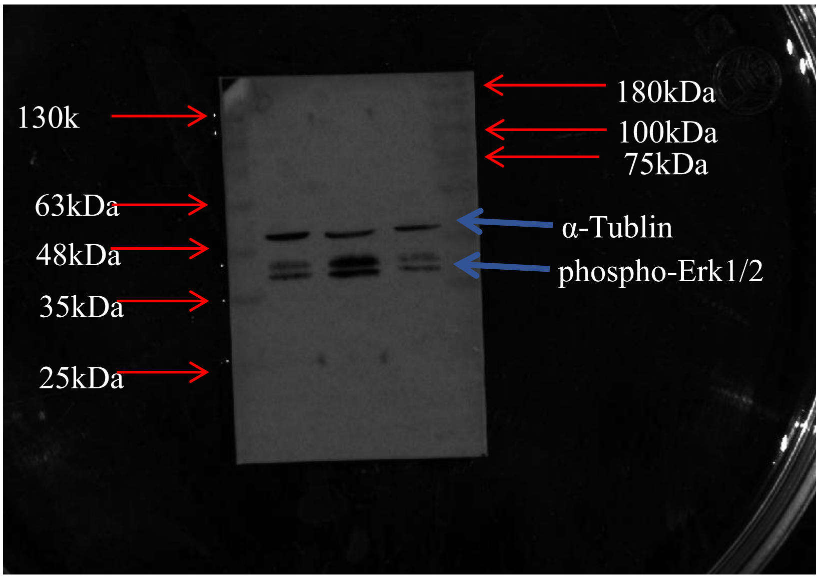

B

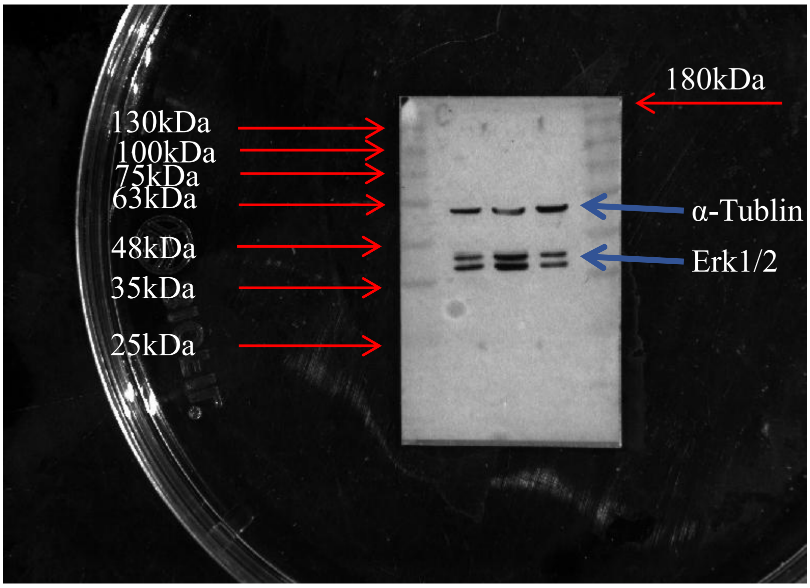

Supplementary Figure 2. The full-length blots involved in Western blot in Figure 3(α-Tublin, phospho-Erk1/2, Erk1/2). (A)The full-length blots of phospho-Erk1/2 protein and came from the same gel. (B)The full-length blots of Erk1/2 protein and came from the same gel. These blots are used in the Figure 3C.

Supplementary Figure 3

A

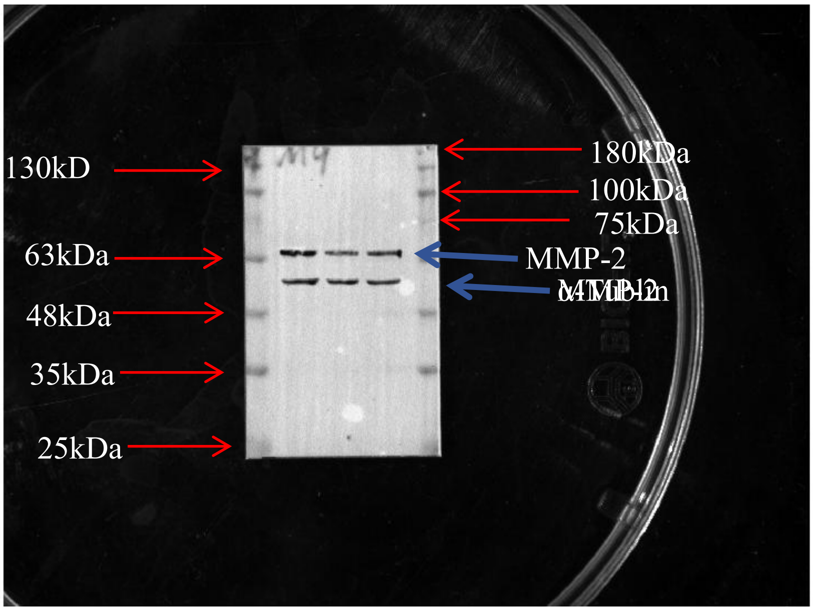

B

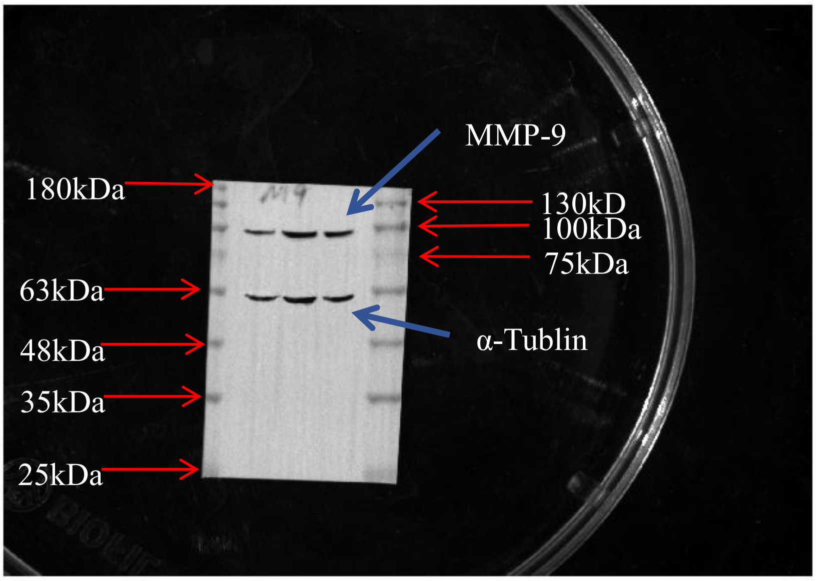

Supplementary Figure 3. The full-length blots involved in Western blot in Figure 4(α-Tublin, MMP-2, MMP-9). (A)The full-length blots of MMP-2 protein and came from the same gel. (B)The full-length blots of MMP-9 protein and came from the same gel. These blots are used in the Figure 4E.

Supplementary Figure 4

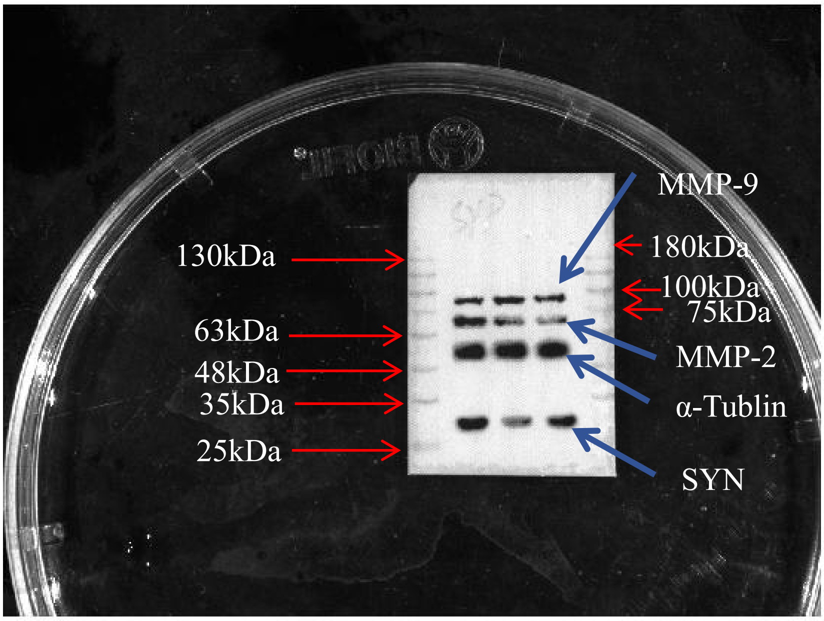

Supplementary Figure 4. The full-length blots involved in Western blot in Figure 5(MMP-9, MMP-2, α-Tublin, SYN). The full-length blots came from the same gel. These blots are used in the Figure 5F.

Supplementary Figure 5

A

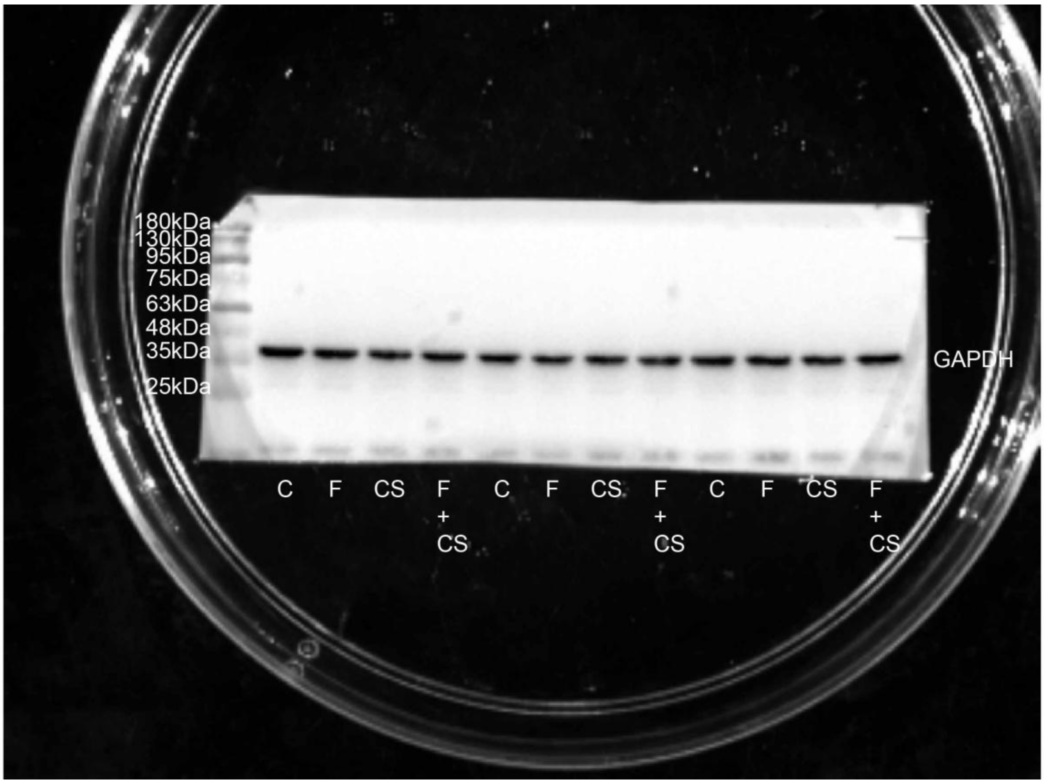

B

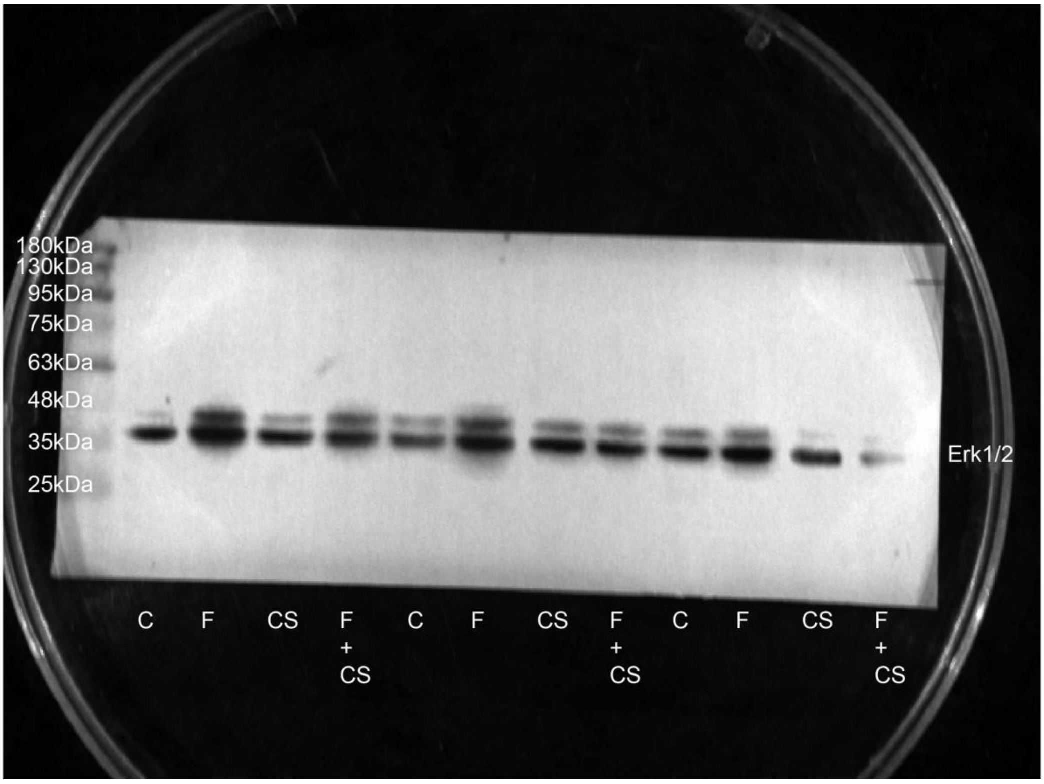

C

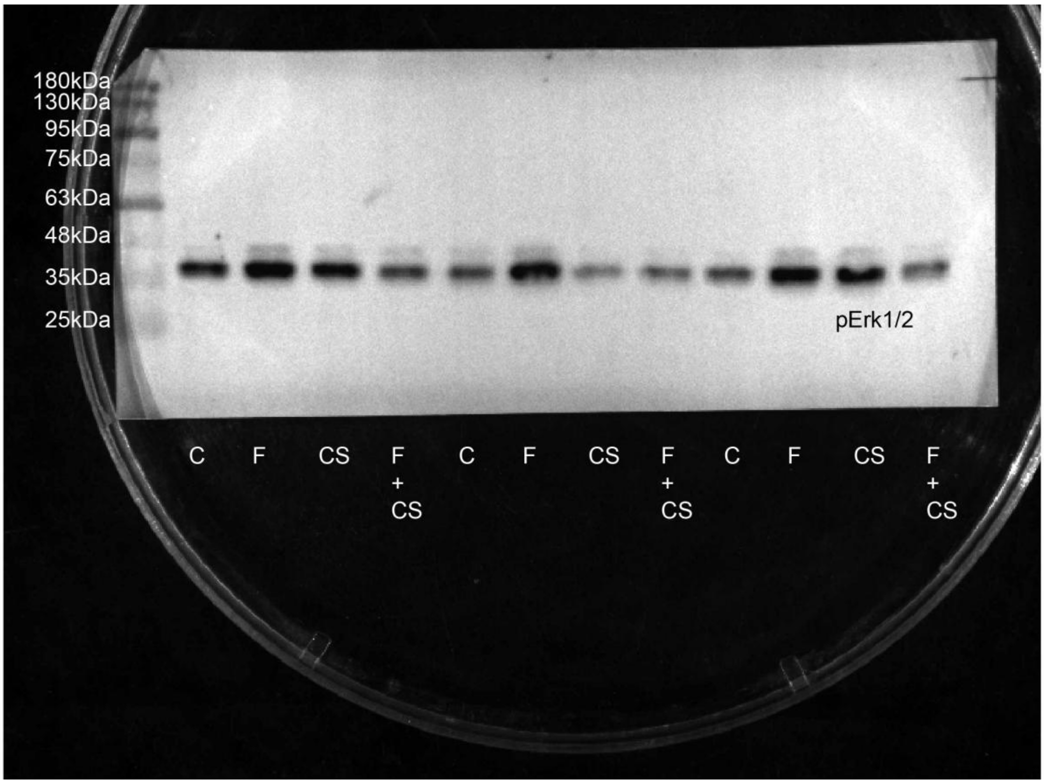
180kDa
130kDa
95kDa
75kDa
63kDa
48kDa
35kDa
25kDa
pErk1/2
C F CS F + CS C F CS F + CS C F CS F + CS

D

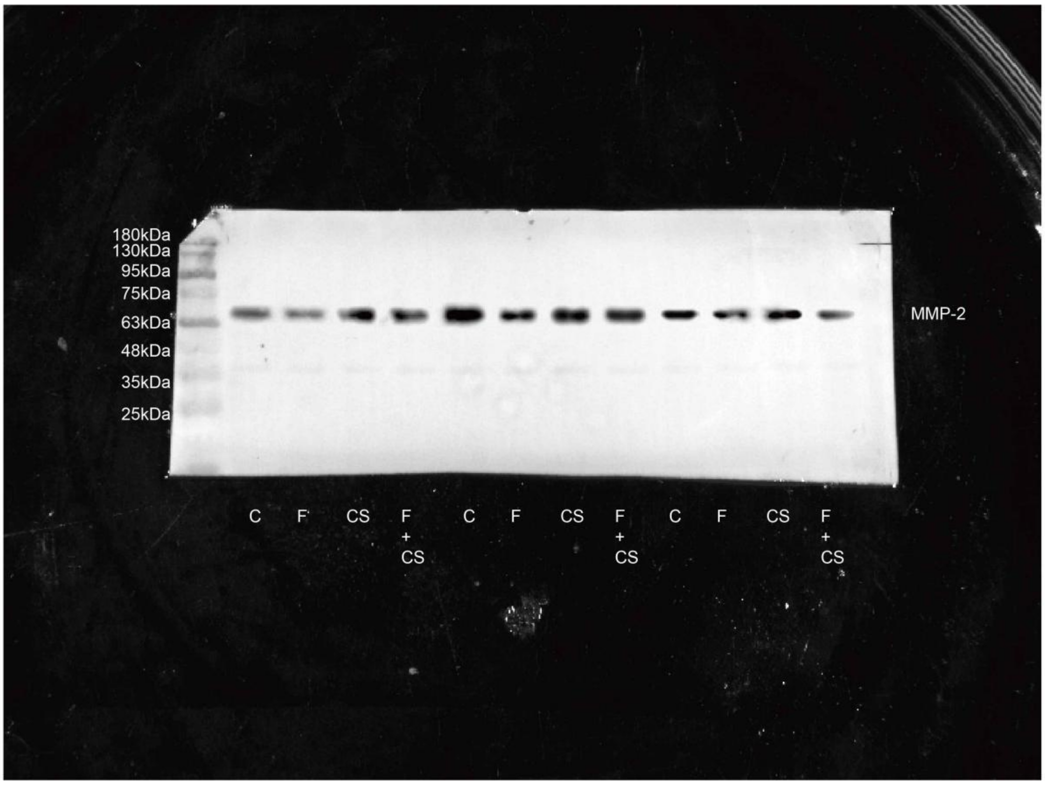
180kDa
130kDa
95kDa
75kDa
63kDa
48kDa
35kDa
25kDa
MMP-2
C F CS F + CS C F CS F + CS C F CS F + CS

E

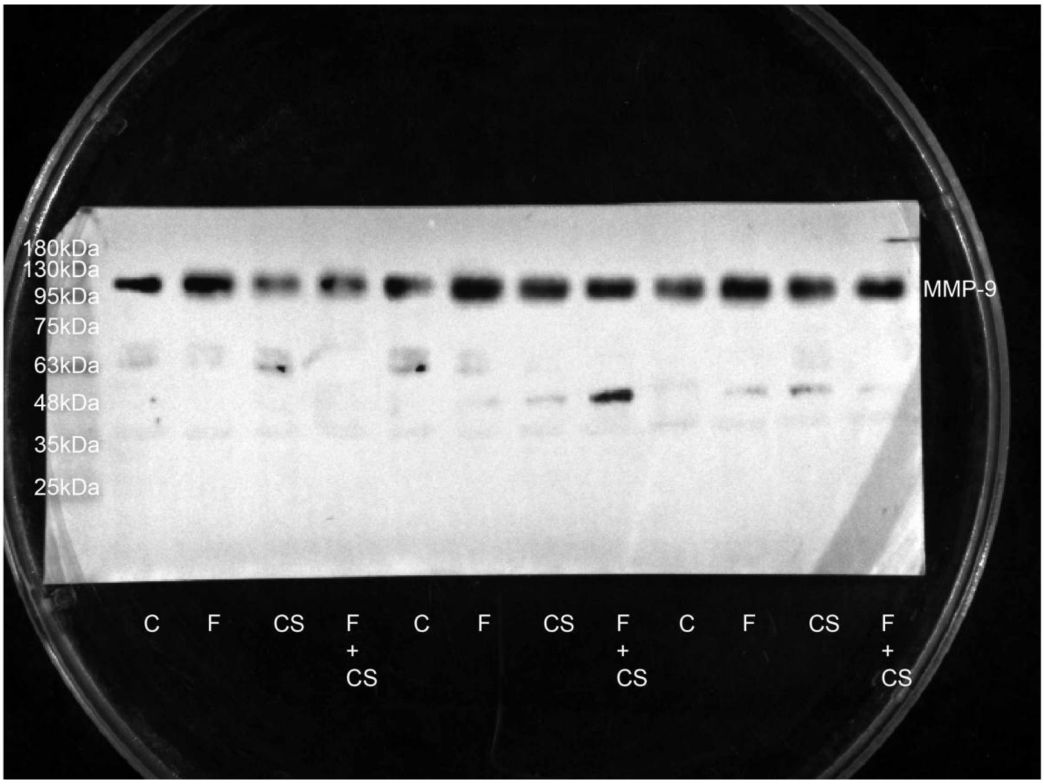
180kDa
130kDa
95kDa
75kDa
63kDa
48kDa
35kDa
25kDa
MMP-9
C F CS F + CS C F CS F + CS C F CS F + CS

F

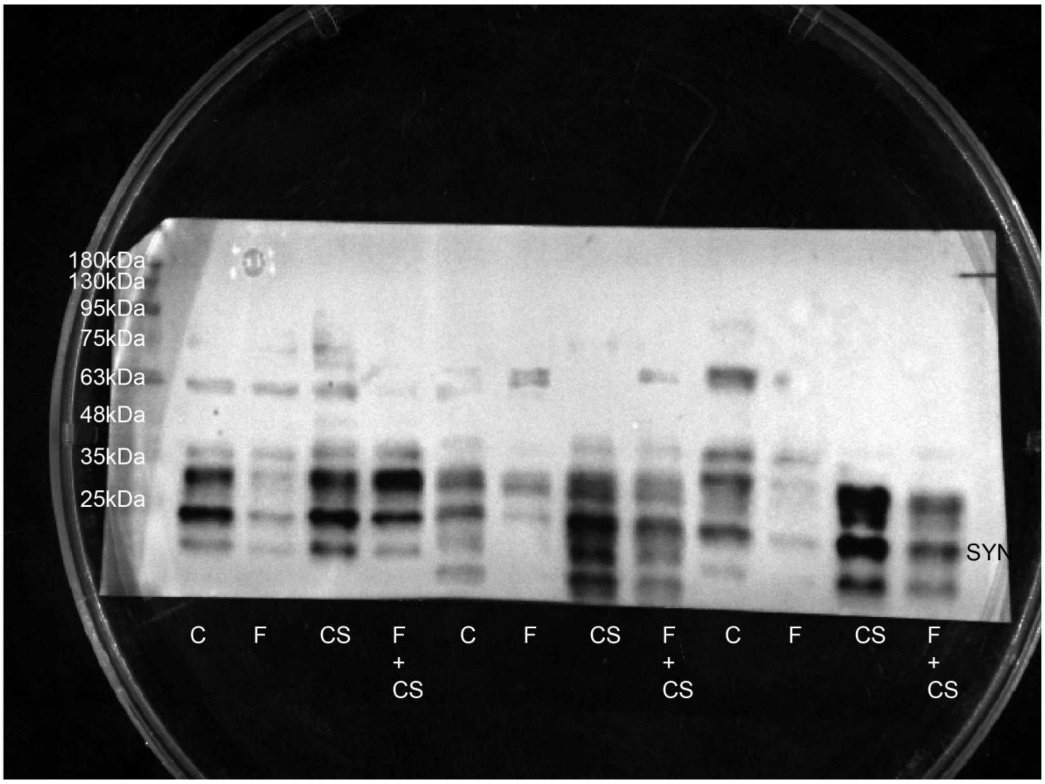
180kDa
130kDa
95kDa
75kDa
63kDa
48kDa
35kDa
25kDa
SYN
C F CS F + CS C F CS F + CS C F CS F + CS

Supplementary Figure 5. The full-length blots involved in Western blot in Figure 4. (A) The full-length blots of GAPDH, (B) Erk1/2, (C) p-Erk1/2, (D) MMP-2, (E) MMP-9 and (F) SYN protein and came from the same gel. These blots are used in the Figure 6C.
